# The Effect of Mixing Milk of Different Species on Chemical, Physicochemical, and Sensory Features of Cheeses: A Review

**DOI:** 10.3390/foods9091309

**Published:** 2020-09-17

**Authors:** Oumayma Boukria, El Mestafa El Hadrami, Sofiane Boudalia, Jasur Safarov, Françoise Leriche, Abderrahmane Aït-Kaddour

**Affiliations:** 1 Applied Organic Chemistry Laboratory, Sciences and Techniques Faculty, Sidi Mohamed Ben Abedallah University, BP 2202 Route d’Immouzer, Fez 30050, Morocco; boukriaoumayma@hotmail.com (O.B.); elmestafa.elhadrami@usmba.ac.ma (E.M.E.H.); 2 Laboratoire de Biologie, Département d’Écologie et Génie de l’Environnement, Faculté des Sciences de la Nature et de la Vie & Sciences de la Terre et l’Univers, Université 8 Mai 1945 Guelma, BP 401, Guelma 24000, Algeria; sofiane.boudalia@hotmail.com; 3 Department of Food Engineering, Faculty of Mechanical Building, Tashkent State Technical University Named after Islam Karimov, University str. 2, Tashkent 100095, Uzbekistan; jasursafarov@yahoo.com; 4 Université Clermont Auvergne, INRA, VetAgro Sup, UMRF, F-63370 Lempdes, France; francoise.leriche@vetagro-sup.fr

**Keywords:** milk mixtures, cheese sensory properties, cheese chemical composition, cow’s milk, camel’s milk, buffalo’s milk, goat’s milk, ewe’s milk

## Abstract

The yield and quality of cheese are associated with the composition, physicochemical, sensory, rheological, and microbiological properties of milk and with the technology applied to the milk before and/or during cheese processing. This review describes the most important research on cheeses obtained from processing mixtures of different milk species and discusses the effect of milk mixtures (i.e., species and mixture ratios) on composition, physicochemical, sensory, rheological, and microbiological properties of cheeses. More specifically, the present review paper will gather and focus only on studies that have provided a clear comparison between cheeses produced from a mixture of two milk species to cheeses produced from only one species.

## 1. Introduction

World milk production largely derives from cattle, buffaloes, goats, sheep, and camels. Among these species, the cow can be considered as the most widespread for milk production. In 2018, the total cow milk (CM) production in the world was around 683 million tons with a population higher than 1.5 billion heads [1]. Buffalo milk production, customary in many countries, ranks second in the world after CM and represents about 13% of worldwide global milk production [2]. In 2018, dairy ewes and dairy goats produced more than 29 million tons of milk [1]. The population of dairy ewes is concentrated around the Mediterranean and Black Sea regions where their dairy products (e.g., cheese) are highly valued by the local population and widely used as typical ingredients in dishes. Dairy goats are concentrated in low-income food-deficit countries of the Indian subcontinent, where their products are an essential food source. Nonetheless, a large population of animals are also found in high-income and technologically developed countries (e.g., France, Spain, and Italy). The total camel population worldwide is around 35 million head with a significant production of about three million tons of milk per year [2].

Milk and dairy products are considered as a potential resource for providing functional foods [3]. This is related to their content of a variety of essential components such as proteins, polyunsaturated fatty acids (FAs), vitamins, minerals, and also to the simplicity of incorporating lactic acid producing bacteria (LAB) during their manufacture. During the last few decades, camel milk (CaM), goat milk (GM), ewe milk (EM), and buffalo milk (BM) have received special attention. This is principally due to their recognition as exhibiting a higher potential for functional foods from a nutritional point of view when compared to CM.

Compared to CM, BM is richer in fat, lactose, protein (especially caseins), vitamins, and minerals [4,5]. More precisely, concerning the lipid fraction, BM is generally associated with higher amounts of saturated fatty acids and lower amounts of unsaturated fatty acids than CM, which contains higher amounts of medium-chain fatty acids (C8:0 to C12:0). Regarding the long-chain fatty acids, BM contained significantly higher contents of myristic acid (C14:0) and palmitic acid (C16:0) and lower contents of stearic acid (C18:0) than CM. BM possesses a higher amount of rumenic acid (C18:2 c9 tr11, the main conjugated linoleic acid; CLA) and total trans fatty acids (C18:1 trans + C18:2 c9 tr11) than CM [6].

GM is generally presented as containing high levels of vitamin A, thiamine, and niacin [7,8] and structural differences in α-lactalbumin and β-lactoglobulin, which are major whey proteins found in CM. The difference in tolerance has also been linked to differences in digestibility rates, aligned with the smaller fat globule distribution of CM. This property is related to the amount of and the structural differences in α-lactalbumin and β-lactoglobulin and to the small-diameter fat globules that allow for higher digestibility compared with CM [9,10,11,12]. Furthermore, GM can be considered as a natural source of lactose-derived oligosaccharides. It presents a healthier lipid composition with increased conjugated-linoleic acid and short FA content and higher vitamins (A and B complex) and Ca^2+^ contents [13,14,15], which means that it may provide a health benefit compared to CM. However, GM also has a high content of saturated FAs and a low content of polyunsaturated FAs, often linked to the development of cardiovascular diseases.

Due to its richness in minerals, higher protein, beneficial fat, and functional bioactive peptides [16], the demand for EM milk is increasing in the global market. It is characterized by the presence of small fat globules with an easily oxidizable membrane. Lipolysis in EM cheeses is faster than in CM cheeses, contributing to an important and typical flavor development [8] due to a higher content of short-chain FAs [17]. EM contains higher protein and fat levels than CM [18]. Compositional differences between EM and CM, mainly in proteins and fats, account for the different technological and sensorial characteristics of cheeses.

CaM presents a high nutritional value and plays a key role in providing milk of superior quality (e.g., more vitamin C, minerals (e.g., K^+^, Cu^2+^ and Mn^2+^), essential and polyunsaturated FAs than CM [19,20]). It is also thought to exhibit properties to manage chronic ailments [21]. The interest in dairy products obtained from CaM has increased in the past decade and the production of CaM on a large commercial scale from modern camel farms is growing [22]. Nowadays, in Mauritania and the United Arab Emirates, many milk and dairy products are produced and marketed (e.g., pasteurized milk, milk powder, fermented liquid milk, and cheese [21,23]). However, the use of CaM in processed food products is very limited and faces difficulties due to the coagulation properties of CaM [24].

In addition to the nutritional functionality that can be provided by these non-cow milks, their properties can contribute to the provision of specific technological functionalities to dairy products (e.g., texture, viscosity, melting, and color). This can be useful for the current consumption trend. Indeed, it is recognized that the modern lifestyle of consumers drives new models of dairy products, especially for cheese consumption (e.g., in fondue and pizza). The nutritional and technological functional properties of these milks and dairy products can participate in producing diversity and catering variations in consumer preferences that can be useful for milk and dairy industries in meeting this demand.

Despite the availability of scientific knowledge about the positive aspects of the consumption of non-cow milk and its derived dairy products [25], its production in some countries is scarce (e.g., Brazil, Morocco, Algeria), limiting its processing into dairy products (e.g., cheeses). This is detrimental because in some regions, mainly rural areas, the valorization in differentiated products may contribute to their economic sustainability [26]. However, for some of the derived products, the flavor of the milk is different; it is stronger than CM, which constrains its acceptability to consumers. In this context, the production of dairy products using mixtures of milk species (e.g., GM with CM) could be an interesting and feasible opportunity for the expansion of the dairy industry in many regions and could equally strengthen the non-CM production chain. Moreover, mixing milk from different species can be a way of improving the quality of fermented dairy products and developing new ones with specific nutritional (biochemical), physicochemical, sensory, and rheological properties. Regarding this opportunity, it is of the utmost importance to characterize the quality features of products derived from mixing different milk species in order to obtain products with proper characteristics and satisfactory acceptance by consumers.

Cheese represents one of the most popular food products in the world. This is probably thanks to its richness in nutritional components like proteins, short-chain FAs, vitamins (e.g., riboflavin, thiamin, vitamin B12), and minerals (e.g., calcium, phosphorus) [27]. The number of scientific studies conducted on the characterization of cheeses produced from mixtures of different species has increased from year to year. The production of 150 papers was observed from 1990 to 2008, while 350 papers were published from 2014 to 2019 (Scopus database on 1 July 2020; keywords: cheese, mixture and milk). Therefore, the present narrative review will gather (Table 1) and focus on the different studies that have provided a clear comparison between cheeses produced from one species of milk (i.e., CM, BM, GM, EM, and CaM) and cheeses produced from mixing different milk species (e.g., CM/GM). Moreover, this review will discuss in detail the effect of milk species and milk ratios on the biochemical (nutritional), physicochemical, structural, sensory, and rheological properties of cheese (Table 1, Table 2 and Table 3). Finally, this paper will present some future research trends in order to improve the knowledge of these types of cheese properties.

## 2. Effect on Biochemical and Physicochemical Cheese Characteristics

### 2.1. Cheese Proximate Composition

Many studies have tried to identify the influence of milk mixtures from different species on the biochemical and physicochemical properties of cheeses. Sant’Ana et al. [29] highlighted that mixing GM and CM in equal proportion (1CM:1GM, volume/volume –v/v-) did not affect the cheese’s protein, moisture, fat, lactose, and ash contents. This corroborates different studies reporting that GM is similar to CM in basic composition [14]. However, other investigations have highlighted contradictory variations in fat content [12,28,30,37]. Some studies have also observed a decrease in protein content [28,30,37], moisture, and salt [30], while others [12] have reported an increase in moisture and fat-in-dry matter (FDM) contents and no significant effects on protein, moisture-in non-fat-substance (MNFS), and salt. According to Ramírez-López and Vélez-Ruiz [28], no differences were observed for lactose and non-fat solids.

It is known that GM micelles have a greater average diameter than CM micelles [41,42] and that its κ-casein exhibits a higher negative charge than in CM [43]. This can participate in dulling the attractive forces between micelles, thereby reducing syneresis during cheese processing, which can modify the final proximate composition of cheese. This was confirmed by previous investigation suggesting that the proportion of coagulable protein and more specifically of αs1-casein, αs2-casein, and β-casein differ between GM and CM. Total and αs1-casein contents were lower and β-and αs2-casein contents were higher in GM milk compared to CM [14,42,44,45]. Moreover, lower rennet coagulation time and weaker rennet strength in GM, when compared to CM, were observed due to differences in composition of individual caseins [8]. Manufacturing differences used to process cheeses (e.g., rennet coagulation properties, ripening temperature, and period) can also lead to a great variation in the final gross composition of cheese [28,30,31].

Concerning the effect of EM when added into CM, Niro et al. [31] in the same study tried to develop Caciocavallo cheeses (i.e., a pasta filata cheese) made with a mixture of CM/EM (4.55CM:1EM, v/v) and CM/GM (1.86CM:1GM, v/v). The authors noted that the percentage of EM added into CM, even when relatively low, caused changes in the nutritional characteristics of the milk and cheeses. The milk analysis demonstrated that mixtures of EM and CM presented the highest fat and protein contents when compared to both the CM/GM mixture and the pure CM. Concerning lactose, the lowest content was reported for CM, whereas milk mixtures presented higher concentrations.

For cheeses, Niro et al. [31] highlighted that the CM/GM mixture had lower fat and higher ash contents than CM cheese. The lower fat concentration was related to differences in biochemical composition of the initial mixed milk and to the processing steps. Indeed, the study reported higher fat loss in the hot water used to stretch the curd due to the smaller fat globules observed in GM. The same observations were reported for lactose and galactose contents among the cheeses because of different microbial activities. These results confirmed the hypothesis that the initial ratio of milk species used to process cheese has an influence on the final cheese biochemical composition.

The production of cheese from CaM is a difficult process under natural conditions due to two main factors: the low level of κ-casein and the large micelle sizes when compared to CM [46]. Usually, after coagulation, CaM gives a weak curd [47,48,49] and a fragile and heterogeneous structure [46] to cheeses. Therefore, different strategies were explored to strengthen the cheese structure, for example, by mixing CaM with bovine milk. Shahein et al. [32] were probably the first team to examine the potential of mixing CaM and BM at different ratios to manufacture soft pickled cheese. The authors reported that increasing the added quantity of BM into CaM increased total solids, fat, and protein contents, while the opposite was noted for moisture and ash. Siddig et al. [34] found equivalent results for a white Sudanese cheese (i.e., Jibna-beida) manufactured by mixing CaM with CM (i.e., 1CaM:1CM, v/v). They also revealed differences in the mineral content of cheeses (i.e., Ca^2+^, Na^+^, and K^+^). Furthermore, the examination of the ash content in mixtures demonstrated an increase when compared to pure CaM cheese. However, protein and lactose levels decreased in cheese containing CM compared to cheese produced with pure CaM.

The study of Siddig et al. [34] further suggested that the final cheese composition was related to the milk coagulation procedure (i.e., coagulation with 10% of citric acid or 5% of a LAB starter culture). When confronted with pure CaM cheese, an increase in fat and total solid contents was observed with LAB coagulation and a decrease was noticed when using citric acid. Regarding mineral content, results indicated a relative increase in Ca^2+^ in the mixture (i.e., 1CaM:1CM cheese) obtained after LAB coagulation compared with those of pure CaM and the milk mixture (1CaM:1CM cheeses) coagulated with citric acid. In contrast, a relative decrease in K^+^ in pure CaM was noted when it was compared with cheeses containing both CaM and CM.

Derar and El-zubeir [22] examined the effect of mixing CaM with EM on soft cheeses. Before cheese making, they noted that the compositional content of CaM was lower than that fortified with EM. In addition, they observed differences between milk varieties and whey produced from CaM, EM, and their milk mixtures. Concerning cheeses, they noted differences in total solid contents between the 1CaM:3EM (v/v) and 3CaM:1EM (v/v) samples. However, protein content showed no difference when compared to standard cheeses (pure CaM cheese). In addition, during storage, differences were found among fat content in cheeses made from CaM, EM, and their mixtures.

The difference observed in the final cheese composition, when a mixture of CaM and CM was manufactured, can be assigned to differences in the initial mixture composition and coagulation properties of CaM and CM. It has been reported that the total solid content of CaM coagulum is lower than that of CM (especially casein; Mehaia [48], Ramet [47]). Moreover, the average size of casein micelles from CaM were larger (200–500 nm) than those from CM (220–300 nm; Farah and Ruegg [46]), while the opposite was observed for the average size of fat globules [50,51]. The size of fat globules and the network formed within the milk fat globule membrane are known to influence cheese yield [52].

The results of the aforementioned studies highlighted that both (i) milk composition (i.e., species origin) and (ii) cheese process manufacturing have an effect on the final cheese proximate composition [53].

### 2.2. Lipolysis and Proteolysis in Cheese

**Figure 1:**
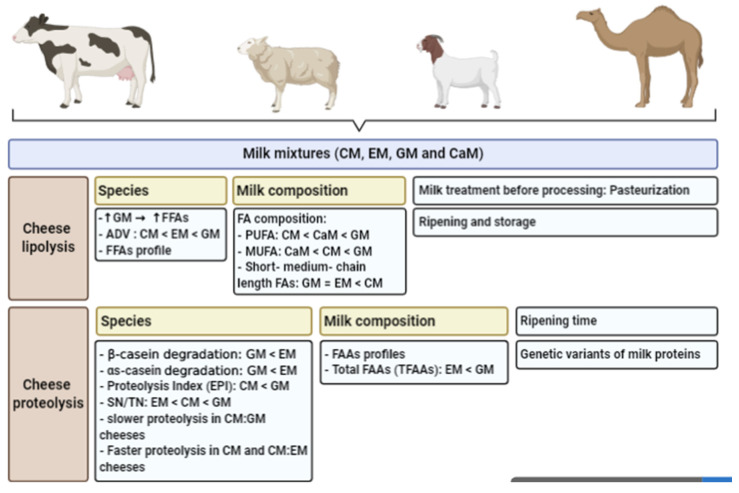
Overview of milk species and milk mixtures on cheese lipolysis and proteolysis. CM: cow milk; EM: ewe milk; GM: goat milk; CaM: camel milk; FAAs: free amino acids; TFAAs: total free amino acids; SN: soluble nitrogen; TN: total nitrogen; FFA: free fatty acids.

Freitas and Malcata [36,39] were likely the first who evaluated the effect of milk mixtures on cheese lipolysis. While comparing Picante cheeses formulated with EM and GM mixtures, they determined that increasing the proportion of GM into cheese milk tended to increase total concentration of FFAs (Figure 1). The same observation was reported by Mallatou et al. [54] in Teleme cheeses (Greek traditional soft) and by Sheehan et al. [12] in semi-hard cheeses. They observed that increasing the proportion of GM into EM above 50% increased the level of FFAs, especially methyl and ethyl esters of short chain acids and terpenes.

In the same way, Queiroga et al. [30] confirmed previous investigations by reporting an increase in total FA content when processing Coalho cheeses from GM, CM, or their mixture (1CM:1GM, v/v).

In the same study, Niro et al. [31] investigated the effect of mixing CM with GM or EM (4.55CM:1EM and 1.86CM:1GM, v/v). As noted by Mallatou et al. [54] in Teleme cheeses, the two formulations had an effect on the ADV factor (defined as FFA content dissolved in a certain amount of fat) depending on the cheese formulation and ripening time. They also noticed that cheese containing EM presented higher lipolysis value compared to the cheeses containing GM.

Concerning the detailed fraction of FFAs, Queiroga et al. [30] identified differences from one cheese to another (i.e., GM, CM, and 1CM:1GM cheeses) particularly for C6:0, C8:0, C10:0, C12:0, C14:0, C16:0, C16:1, and C18:2n6c. GM and 1CM:1GM (v/v) cheeses showed higher contents of short- and medium-chain FAs (e.g., C6:0, C8:0, and C10:0). Higher amounts of C12:0 in GM cheese were found only after 28 days of storage while CM cheese presented higher amounts of C16:0 and C16:1 than GM and 1CM:1GM (v/v) cheeses at the end of the storage time. Concerning the C18:2n6c, the highest amount was found in cheeses containing GM when compared to CM. Equivalent findings have been reported by different authors [29,53,55,56].

The differences observed in lipolysis and FFA content in cheeses can be related to the initial FA composition of milk due to the different reactions of mammalians (e.g., goat, cow, and camel) to dietary factors both in fat secretion and in the FA composition of milk [57]. These different responses are dependent on the metabolism and biohydrogenation of FAs in the rumen obtained from feeding cattle species [58]. Moreover, a higher saturation of the biohydrogenation of dietary FAs in the rumen of these animals can result in a difference in FAs production (e.g., lower or higher production of long-chain unsaturated FAs) [55]. For example, CM and GM exhibited 70% of SFA in fat content while the level of SFA in camel was 62.5% [59]. The levels of PUFA in CM, GM, and CaM fat were 2.9%, 4.7%, and 3.9%, while that of MUFAs were 27.7%, 24.5%, and 42.6%, respectively [60,61,62]. GM and EM fat also present a significantly higher level of short and medium chain length FAs (C6:0–C14:0) than CM [14,41,45,63,64]. The different conditions applied to milk before processing (i.e., pasteurization) and for ripening and storage (temperatures, salt concentration of brine …) of cheeses may affect the lipase activity [65,66,67,68,69], which principally governs the lipolysis action during ripening.

Regarding proteolysis, Freitas and Malcata [70] noted that milk type affects the proteolysis reaction in Picante da Beira Baixa cheese (i.e., hard, spicy, salty traditional Portuguese cheese). Nonetheless, the observed differences were probably a consequence of higher initial contamination of GM. Freitas and his collaborators [36,40] noted that the extent of hydrolysis of αs2- and β-caseins varied with milk composition. In general, a higher GM fraction led to higher levels of γ-caseins, [40] derived from the breakdown of β-casein. The degradation of β-casein by 180 days was 18.8, 26.4, 40.2, 55.6, and 36.5%, for cheeses manufactured with 0, 25, 50, 75, and 100% (v/v) GM into EM, respectively; while degradation of αs-casein was 35.9, 40.2, 81.8, 93.0, and 68.7%, respectively. These observations were confirmed one year later by the same team [71]. Conversely, Mallatou et al. [72] reported, during the processing of Teleme cheeses with EM and GM, an inverse trend concerning the degradation level of αs-casein compared to β-casein. In their study, about 60% of the αs-casein originally present was degraded in cheeses made from EM or a mixture of 1EM:1GM (v/v) milk by 360 days of aging, but only 36% of the αs-casein was degraded in cheeses made from GM over the same period. However, about 40%, 28%, and 36%, respectively, of the β-casein originally present was degraded in the cheeses made from EM, GM, and a mixture of EM and GM at 360 days of ripening.

Furthermore, other authors have described the proteolysis effect after mixing milk by combining other mammalian species, especially with CM, for example, the study performed on Minas fresh cheese during cold storage conducted by Sant’Ana et al. [29]. During the study, they concluded that the extent of proteolysis index (EPI) observed for pure CM cheese was lower than for 1CM:1GM (v/v) and GM cheeses (Figure 1). Nevertheless, the presence of small- and medium-chain peptides and FAAs evaluated by the DPI factor (depth of the proteolysis index) was not different between cheeses. In the same way, Niro et al. [31] observed significant differences in the primary (SN/TN) and secondary proteolysis (NPN/TN) among Caciocavaillo cheeses made from a mixture of 4.55CM:1EM (v/v) and 1.86CM:1GM (v/v), confirming results obtained by Molina et al. [73]. These authors asserted that the highest concentrations of the SN/TN ratio were recorded in cheeses made from GM (i.e., 1.86CM:1GM, v/v) followed by CM cheeses, and finally by cheeses containing EM (i.e., 4.55CM:1EM and EM cheeses). Concerning the NPN/TN index, an increase was noted during and at the end of ripening in 1.86CM:1GM cheeses compared to CM and 4.55CM:1EM cheeses. The different behavior found highlighted slower proteolysis in 1.86CM:1GM cheeses and faster proteolysis in CM and 4.55CM:1EM cheeses. These results were confirmed by the work of Imm et al. [74], who compared proteolysis in CM and GM Mozzarella cheeses during storage.

From the literature, studies concerning FAA profiles revealed significant differences according to the relative proportions of EM and GM. Freitas et al. [40] noted that valine changed from 251.79 ± 0.99 to 352.20 ± 16.49 mg/100 gDM during ripening (at 140 days) when CM was replaced by GM in the mixture. One year later, the same authors [71] reported that cheeses manufactured with 20% (v/v) GM milk exhibited the highest content of FAAs. Finally, Freitas and Malcata [39] concluded that 50–80% (v/v) GM maximized proteolysis in Picante da Beira Baixa cheese. However, a statistical analysis (ANOVA) revealed that ripening time has a higher effect compared to the initial milk composition [71]. Niro et al. [31] also evaluated the detailed analysis of the TFAA and FAA profiles of Caciocavallo cheese made from a mixture of CM and EM. First, they noted an increase in TFAA in all cheeses. In general, the highest TFAA content was found in pure CM cheeses, and the lowest in 1.86CM:1GM cheeses. Second, concerning the profile of the FAAs, they reported that all cheeses exhibited an increase in individual FAAs except for arginine during ripening. Tyrosine and histidine were found only in mixed CM and EM cheeses (4.55CM:1EM). Equivalent results were reported in Teleme cheeses made from a mixture of GM and EM [54,75], in Feta [75], and in Turkish white cheeses made from pure EM [76].

Contrary to previously cited studies, in 2009, Sheehan et al. [11] reported contradictory results on semi-hard cheeses manufactured from a mixture of GM and CM. They noted that substitution with up to 50% CM had no significant effect on primary proteolysis and that substitution of up to 75% CM had no significant effect on the levels of total or individual FAAs.

As reported by Mallatou et al. [72], the low degree of proteolysis generally reported for cheeses containing GM may be due to the inaccessibility of enzymes from different sources (rennin, LAB, non-starter LAB, and possibly plasmin) to hydrolyze the specific bonds of caseins. In addition, it is known that the variations in different genetic variants of milk proteins in GM and EM may explain the variances in the degree of proteolysis of cheeses made from different types of milk [77]. Pierre et al. [78] demonstrated that the composition and the physicochemical characteristics of GM were different when containing the A or O αs-casein variants. Trujillo et al. [79] also found that variant A was hydrolyzed faster than variant F and the proteolytic pattern differed between the variants in many young GM.

### 2.3. Acidity and pH of Cheese

Most of the studies concerning the effect of milk mixtures on the pH of cheese were carried out by using GM, EM, and CM, while a lower proportion of studies were performed with CaM. Sheehan et al. [12] performed one of the first studies demonstrating that increasing the proportion of CM (i.e., pure GM, 3GM:1CM, 1GM:1CM, 1GM:3CM, pure CM) in the cheese milk resulted in increased curd pH. Nevertheless, they observed that substitution of up to 75% CM for GM (i.e., 1GM:3CM) had no significant effect on the pH of cheese during ripening. Queiroga et al. [30] confirmed these findings later and demonstrated that mixing GM and CM resulted in equivalent pH values for their derived fresh cheeses during ripening. These observations contradict the investigation of Ramírez-López and Vélez-Ruiz [28], who reported that the pH values of the cheeses manufactured from milk blends (1GM:9CM, 1GM:4CM, 1GM:1.5CM, 1GM:1.5CM, v/v) were greater than the pH of the control (i.e., pure CM cheese). This effect was explained by the higher alkalinity and buffering capacity of GM compared to CM [8].

Additionally, Niro et al. [31] investigated the effect on the pH of mixing CM with EM or GM in the processing of Caciocavallo cheeses. The authors showed a slight pH decrease and a consequent increase in acidity values for all the cheese formulations (i.e., pure CM, 4.55CM:1EM and 1.86CM:1GM, v/v). The 4.55CM:1EM cheeses presented the highest acidity compared to pure CM and 1.86CM:1GM cheeses. Concerning pH values, the 1.86CM:1GM cheeses presented the highest values during ripening when compared to pure CM and 4.55CM:1EM cheeses. The lowest pH values were reported for the cheeses containing EM (4.55CM:1EM).

In another approach, Mallatou et al. [72], evaluated the effect of mixing GM and EM in equal proportion on the pH of Teleme cheese during ripening. They observed only significant differences on day 1 of ripening between GM and 1EM:1GM cheeses, while no difference was observed when compared to EM.

Regarding CaM, Derar and El-Zubeir [33] investigated the compositional properties of soft cheese made from CaM, EM, and their mixtures (3CaM:1EM, 1CaM:1EM, 1CaM:3EM). The pH and acidity were evaluated during storage (37–40 °C for during 21 days). On day 1, the highest acidity value was noted for the pure EM cheeses and decreased with decreasing the proportion of EM in the mixture (1CaM:3EM > 1CaM:1EM > 3CaM:1EM). These differences in acidity were maintained during storage. Moreover, an increase in acidity was previously noted for five different types of cheeses prepared from CaM [49,80]. This increase was attributed to the growth of bacteria in the cheese during the storage period [22] and indicated that the development of acidity was accelerated after adding 10% EM, which reduced the buffering capacity of the milk compared to CaM alone [47]. These results contradicted those of Siddig et al. [34] when CaM was mixed with CM. The authors noted that cheeses containing CaM and CM (1CaM:1CM) presented the lowest pH when compared to pure CM cheese. The findings of Siddig et al. [34] fully match the results of Yonas et al. [81] and Haider et al. [82].

## 3. Sensory and Rheology Features of Cheese

Mallatou et al. [35] reported what are probably the first results concerning the effect of mixing milk from different species on the sensory properties of cheese. During the processing of Feta cheese, they noted that pure EM cheese had the highest scores in terms of body-texture than the other cheeses produced by mixing EM and GM. Statistical analysis showed that the force necessary to cause fracture in cheese samples made from pure GM was higher than the samples made from 50% GM (1GM:1EM), 25% GM (1GM:3EM), and 100% EM. This suggested that the softest cheeses were found to be pure EM and the hardest ones were those of pure GM. Freitas et al. [40] reported contradictory results. Indeed, they claimed that the volumetric ratio of EM to GM milk was not statistically significant in terms of its effect upon surface, form, and texture. However, contrary to Mallatou et al. [35], they observed that cheeses manufactured with either 75% of GM (1EM:3GM, v/v) or pure GM received the best scores for texture. Regarding Minas fresh cheese processes from CM and GM, Sant’Ana and coworkers [29] also noted that the 1CM:1GM (v/v) cheese displayed a difference only at the beginning of storage (day 1) when compared with the other cheeses (pure CM and pure GM). When the analysis of the texture profile (TPA) was investigated, the authors noted that the type of milk used to manufacture cheese and the length of storage did not affect the adhesiveness, elasticity, or cohesiveness of the cheeses. Nonetheless, the CM and 1CM:1GM (v/v) cheeses displayed decreased gumminess and chewiness values over the storage period evaluated, whereas this behavior was not observed in GM cheeses.

Niro et al. [31] reported the comparison between the effects of adding EM or GM into CM milk on cheese texture properties. During their investigation, they managed three pasta filata cheese productions made with 4.55CM:1EM (v/v), 1.86CM:1GM (v/v), and pure CM and noted higher scores for elasticity and adhesiveness of the pure CM cheeses. Moreover, higher friability was observed for the cheeses containing EM, corroborating the investigations of Mallatou et al. [35] and Tsigkros et al. [83].

In a different approach, Shahein et al. [32] explored the potential for mixing CaM and BM on the body/texture of Domiati cheese. The authors studied five ratios (9CaM:1BM, 4CaM:1BM, 2.33CaM:1BM, and 1.5CaM:1BM, v/v) and their respective pure milks (pure CaM and pure BM). As noted for mixtures containing CM, the authors observed that adding BM improved the body/texture of cheeses after 45 days of pickling. These results are in agreement with those reported by Shahein et al. [32].

The differences in the cheese texture parameters observed by the aforementioned studies can be attributable to different factors: the initial composition of milk and cheese (fat, protein and moisture), the manufacturing process (brining, dry salting), and the extent of the proteolysis index [84]. For example, it has been shown that high acidity, protein, and total solids content generally make cheese harder and less easily deformed [85,86]. Conversely, a high-moisture-content of cheese is associated with a fragile protein network that results in less-firm cheeses [87] and a higher degree of unsaturation of FAs is correlated with a smoother texture [88]. Despite these different factors and regardless of dairy species (i.e., cow, goat, and sheep), casein gels are responsible for most of the various rheological/textural properties of cheese, stretch and fracture [89]. It has been shown that smaller micelles form a more compact and, hence, firmer gel network than larger micelles [90,91], whereas contradictory results have been obtained for rennet clotting time [92,93]. Concerning the differences observed between the sample containing EM and CM, these disparities might be due to different casein structures or concentrations in the milk, where ovine milk contains higher levels of casein than caprine milk [94]. GM differs from CM in several physicochemical characteristics, which explains major differences in the technological behavior of the two types of milk [95]. The poorer cheese making ability of GM is largely attributable to the lower casein content, and to specific properties of casein micelles in GM such as their composition, size, and hydration [95]. GM also has different proportions of the four major caseins compared to its CM counter-parts, and there are great variations, especially between αs1-casein and αs2-casein content between individuals and breeds of goats and sheep due to the occurrence of genetic polymorphisms of all milk proteins, which greatly influence their cheese making properties [95].

Concerning flavor properties, different authors have investigated the effect of mixing milk from different species on this important cheese feature. From this perspective, Queiroga et al. [30] studied the effect on the sensory features of Coahlo cheeses obtained after mixing CM and GM. The authors reported that increasing the proportion of the cheese milk to 50%, bovine had no significant effect on the fruity flavors that are associated with CM cheeses. However, waxy/goaty and bitter flavors were present at lower intensities than in cheeses manufactured from 100% GM. Sheehan et al. [12], on semi-hard cheeses, confirmed these previous investigations and associated the decrease in goaty flavor mainly to a reduction of flavor compounds such as methyl and ethyl esters of short chain acids and terpene. Sant’Ana et al. [29] also highlighted in Minas fresh cheese that the higher short-chain FA contents promote the typical aroma and flavor of the dairy products made with GM [96]. Those conclusions were reported after having compared cheeses formulated with 1CM:1GM (v/v) and cheeses containing only CM or GM. Nonetheless, they observed that the three categories of cheeses did not differ in their salty and acidic flavor. The CM cheese was described as exhibiting a better wet appearance compared with cheeses containing GM (pure GM and 1CM:1GM (v/v) cheeses). This difference in the wet appearance corroborated the higher syneresis observed by the authors during CM processing. CM cheese received higher scores for butter flavor and aroma, whereas GM cheeses received higher scores for global aroma and flavor when compared with CM and CM:GM cheeses. These results matched those of Niro et al. [31]. During their investigations, they noted higher scores regarding sweet attributes for pasta filata manufactured from pure CM compared to cheeses produced from mixing CM and EM or CM and GM. In the same study, Niro and coworkers claimed that samples containing CM and EM (4.55CM:1EM) displayed higher scores for intensity of flavor, acidic, astringent, and salty attributes after 30 days of ripening. In contrast, samples containing a mixture of GM and CM were found to exhibit high solubility, together with the intensity of aroma and bitter attributes. Moreover, CM cheeses presented higher scores regarding sweet attributes when compared to other cheeses.

The conclusions above-mentioned are in contradiction with the investigations of Freitas et al. [71]. Indeed, the researchers reported, after the analysis of 1GM:1EM, 1GM:3EM, and 3GM:1EM (v/v) cheese formulations, that the volumetric ratio of EM to GM milk was not statistically significant in terms of its effect upon flavor and overall sensory parameters of cheeses. However, contrary to Mallatou et al. [35], they observed that cheeses manufactured with either 75% GM (1EM:3GM, v/v) or pure GM, and ripened for 180 days received the best scores for flavor. Moreover, they observed that cheeses made from pure GM exhibited the second highest score, while the other cheeses had no significant differences regarding flavor. Even when cheeses were made from pure GM, no panelists detected a goaty flavor, contrary to the study of Queiroga et al. [30]. However, no significant difference between pure GM and cheeses containing 75% GM (3GM:1EM) was reported. As the storage period was prolonged from 60 to 120 days, the authors noted that the sensory quality of Feta cheeses produced from GM deteriorated, and, in contrast, that made with EM improved.

When considering camelids, Siddig et al. [34] reported that mixing CaM with CM (i.e., 1CaM:1CM) to produce Jibna-beida cheese did not decrease the acceptability of the final products. All of the cheeses were highly rated by the panelists with preference to cheese prepared by using starter culture, which was better received compared to that prepared by an acidification process. It can be concluded that production of an acceptable quality Jibna-beida cheese from a mixture of CaM and CM is feasible.

Concerning color, most of the studies reported the effect of mixing different milk species on the whiteness, yellowness, and redness of cheeses after analysis by a sensory panel or physical measurements (e.g., chromameter). In this context, Queiroga et al. [30] depicted that Coahlo cheeses made from 1CM:1GM (v/v/) and pure GM presented higher L* (white component) values from seven days of storage onward. These results were in contradiction with Sheehan et al. [12], who reported no effect of milk type on cheese whiteness. Nonetheless, cheeses made from GM are generally whiter in color [53]. This is related to the fact that goats can convert β-carotene into vitamin A and also produce milk with smaller-diameter fat globules compared to that produced by cows [14,55]. Moreover, Álvarez et al. [97] observed a positive correlation between the moisture content and L* in cheeses made with GM, suggesting that a high moisture content results in lighter products. Higher a* values (red component) were found in GM cheeses. According to Sheehan et al. [12] the increase in a* value in cheeses is directly related to the addition of GM and more specifically to their FA profiles. The b* values (yellow component) were found to be higher for CM cheeses. This difference in color is assigned to a high-level transfer in cows of carotenoids from their diet to milk and, consequently, dairy products made with CM are more yellow than those prepared with milk from other species [98]. These results corroborated Sant’Ana et al. [29] and Ramírez-López and Vélez-Ruiz [28] on Minas fresh cheese (GM, CM and an equal mixture of both) and Fresh Panela cheeses (1GM:9CM, 1GM:4CM, 1GM:2.5CM, 1GM:1.5CM, v/v), respectively.

For cheeses processed from CaM, Shahein et al. [32] noted no difference in appearance/color on Domiati cheeses (soft pickled cheese) made from different mixtures of CaM and BM (9CaM:1BM, 4CaM:1BM, 2.33CaM:1BM, and 1.5CaM:1BM, v/v) and their respective pure milks (pure CaM and pure BM) during storage. These results are in agreement with that reported by Shahein et al. [32].

## 4. Microbial Ecosystems of Cheese

Milk microbial ecosystems are the main agents that contribute, via their metabolic activity, to the quality of dairy products in terms of flavors, aroma, and texture, but also with regard to safety. Numerous intrinsic and extrinsic factors drive the composition and richness of these communities, among them farm practices of dairy herd management, feed quality, season, stage of lactation, animal health, weather conditions, water quality, and hygienic practices of the milking stage are the most frequently described [99]. In this sense, so-called special composition milks are particularly relevant models. More than bacteria present in milk at a given time, what matters is the expression of their metabolic functions together with their interactions. Indeed, structure and organization of the bacterial community is determined by the chemical composition of milk and, more precisely, the nutritional content and accessibility, and the presence or not of immunological factors and other antimicrobial agents.

All the studies converge to show that the microbial loads and composition of raw milk before any treatment vary considerably between geographically distant farms, among herds from the same region and even among samples from the same animal or herd. Accordingly, considering these variations, the comparison of published data about the microbial composition of milk from different species all over the world can therefore be misleading. Moreover, from culture-based enumeration and phenotypic identification methods to high-throughput sequencing approaches, the information gathered also differs. The first aims at the quantification of taxonomic or functional microbial “groups” that are able to grow on rich and/or selective media. The second, which is based on culture-independent DNA-based technologies detects dead cells as well as extracellular DNA and viable populations. As explained by Metzger et al. [100], in milk, bacterial DNA originates mainly from the animal’s skin or environment, from the keratin of the teat canal, from leukocytes in the milk, and milk within the mammary gland environment. Accordingly, one can assume that at the tank level, the presence and diversity of bacterial populations relies mainly on the way that the milking process favors or limits the transfer of bacteria to raw milk. Nevertheless, several authors have managed the description of milk microbiota from different occidental animals (cow, goat, sheep, buffalo) [101] and identified that most of the bacteria belongs to four main phyla: *Proteobacteria, Actinobacteria, Firmicutes*, and *Bacteroidetes*. At the genus level, *Pseudomonas* spp. are often predominant but its environmental origin suggests a link with milking hygiene more than the animal itself. LAB such as *Enterococcus*, *Lactobacillus*, *Lactococcus*, *Streptococcus*, *Leuconostoc*, *Pediococcus*, and *Weissella* are omnipresent, but their proportions vary greatly. According to the sample origin, a great diversity of other bacteria detected in low abundance is often observed.

Considering mixtures of milk from different animal species, it is likely that at the time of mixing, total microbial composition would be the simple addition of microorganisms from each milk. However, a few minutes later and during the subsequent transformations from milk to cheese or other dairy products, the questioning is different. The multiple and dynamic variations of biotic and abiotic factors would determine the metabolic activity of bacteria, yeast, and molds as well as their interactions. Indeed, the different strains might have unequal levels of adaptability after contact with the components of their non-native milk varieties or changes during the cheese-production process. All around the world, numerous traditional cheeses or dairy products are manufactured from ruminant-milk mixtures. In most cases, these practices aim to overcome the low-volume of production of each herd and are the fact of small or very small dairy farms with poor hygienic practices. These studies put more emphasis on the spoilage and pathogen microflora (i.e., *Pseudomonas* spp., *Bacillus* spp., *Clostridium* spp., *Salmonella*, *Listeria monocytogenes*, *Escherichia coli* 0157:H7, and *Staphylococcus*) and the way to control them including the diversity and antimicrobial properties of their LAB population (for review, see Alexandraki et al. [102]). Nowadays, industrialists from the dairy sector seek to develop new products exploiting the nutritional qualities of non-cow milks with the double aim to promote local resources and to fit the consumer demand. These products may include drinking milk, yoghurt, butter, and different types of cheese.

In 2014, Niro and collaborators presented innovative pasta filata cheeses made from a mixture of raw CM with EM (4.55CM:1EM) or GM (1.86CM:1GM). The process was similar to that of Caciocavallo, which consists of the addition of mesophilic and thermophilic LAB, followed by the addition of rennet in milk. In fact, the particularity of this cheese lies in the fact that curd formation and kneading are performed in hot water (80 °C) before salting and ripening for 2 months at 10 °C. Mixed CM:EM and mixed CM:GM cheeses presented higher mesophilic rod and cocci LAB counts in comparison with CM cheeses during the 60 days they were tested, but the latter exhibited a higher presence of thermophilic bacteria over the same period; it should be noted that the differences never exceeded 0.5 log. In all cheeses, the fecal coliforms were not detectable at 60 days of ripening. Moreover, total coliforms, *enterobacteria*, and molds were undetectable or stayed at very low levels in all cheeses.

## 5. Conclusions: Limitations and Future Trends

This review documents the current impact of mixing different milk species on cheese attributes (e.g., proximate composition, sensory features). Based on the above studies, it has been fully demonstrated that the knowledge of how (i) the animal species from which the milk originates and (ii) the proportions of each species used in the mixture influences the quality of cheese, is necessary to design products with improved physicochemical, nutritional, functional, and sensory qualities. Even though extensive research has been conducted on milk blends and their use in cheese production, many other characteristics of these products need to be investigated. For instance, it is evident that much attention has to be devoted to the microstructure, microbiology, and release of bioactive compounds in cheese obtained from milk mixtures. These three factors are of the utmost importance and require an appropriate understanding of their relation to the quality of cheese and dairy products in order to design bespoke products.

Therefore, future studies on milk blends from different species for cheese production could focus on these three factors. Indeed, the microstructure of dairy products is one of the major determining factors of texture, physicochemical properties, flavor, color, nutritional profile, and bioavailability of nutrients among other characteristics [103]. The understanding of the impact of the association of milk from different species on molecular structure, location, and interactions of components (e.g., fat, proteins) during manufacture is essential for predicting the quality attributes of dairy products, particularly cheese. Different techniques are available nowadays to explore the microstructure of dairy products in detail (e.g., computer tomography, x-ray imaging, magnetic resonance imaging). For more information, one may refer to the recent review published by Lei and Sun [104].

Moreover, concerning microorganisms, as far as we know, the microbiota of CaM has not yet been sequenced and conventional agar plate enumeration did not show the existence of a specific species, so there is a gap to fill. However, numerous studies have reported the presence of Lactobacillus species able to tolerate the condition of the gastrointestinal tract, while exhibiting antibacterial activity against bacterial pathogens. These strains could be good candidates as starters or probiotics [105]. Moreover, most research around non-cow milk has focused on the characterization of a few species that could present specific characteristics such as probiotic value or starter qualities [106,107]. It will be able to rely on the potentialities of metatranscriptomics and metabolomics techniques to understand the interactions between the microorganisms and the components of the dairy matrix because the release of bioactive compounds is closely related to the composition of dairy products.

Concerning bioactive compounds, it has been reported in the present review paper that some studies have focused their attention on peptides and FAs. Nonetheless, several compounds, like GABA, conjugated linoleic acid (CLA), vitamins, and exo-polysaccharides can be found in dairy products. Those compounds play an important role in both the development of the texture and in the flavor of cheese. At the same time, they can exhibit interesting antioxidant, antimicrobial, immunological properties, and show potential in disease prevention (e.g., obesity, dyslipidemia, and type 2 diabetes). These compounds can be naturally present in milk while others can be released by microorganisms during processing. For example, microorganisms like LAB can release several molecules in milk during cheese ripening (e.g., vitamins, exo-polysaccharides). Therefore, the identification of those bioactive compounds is of primary importance, as is their characterization via metabolomics, separation, and detection techniques.

Finally, we assumed that the development of appropriate technologies for the production of innovative cheeses using a mixture of cow, ewe, and goat milks among others, with proper characteristics and satisfactory acceptance by consumers, could be an interesting and feasible opportunity for the dairy industry, allowing its expansion in the market. It is also evident that special attention should be paid to applied and innovative research to adapt to the different non-cow milk sectors to future production and market trends.

## Figures and Tables

**Table 1:** A summary overview of the different studies conducted on cheeses ^1^.

Product	Country	Milk Mixture	Milk Ratios Studied	Breed	Coagulation	Ripening/Storage	Weight	Cheese Shape/Mold Shape	Reference
Fresh Panela cheese	Mexico	CM and GM	9CM:1GM, 1CM:1GM, 1.5CM:1GM, 1CM:1.5GM, 1CM:9GM (v/v) ^1^	-	CHY-MAX and 10% CaCl_2_	15 days 4 °C	280 g	Molds (14.5 × 10.5 × 0.8 cm)	Ramirez-Lopez and Vélez-Ruiz [28]
Minas Fresh cheese	Brazil	CM and GM	Pure CM, pure GM and 1CM:1GM (v/v) ^1^	Cow (Girolando breed) Goat (Alpine breed)	Starter culture DVS (R-704 *Lactococcus lactis ssp. lactis* and *Lactococcus lactis ssp. Cremoris* calcium chloride (0.5 mL/L) and 0.8 mL/L of commercial rennet (Hala)	21 days at 4 °C	-	-	Sant’Ana et al. [29]
Coalho cheese	Brazilian cheese	CM and EM	1CM:1GM (v/v) ^1^	-	Direct acidification (0.25 mL/L lactic acid), Calcium Chloride (0.5 mL/L), commercial rennet 0.9 mL/L HaLa, starter mesophilic lactic cultures (R-704 *Lactococcus lactis subsp. Cremoris and L. lactis subsp. Lactis*)	28 days at 4 °C	250 g	Rectangular container	Queiroga et al. [30]
Semi-hard cheese	-	CM and GM	Pure CM, pure GM, 3CM:1GM, 1CM:1GM and 1CM:3GM (v/v) ^1^	-	0.15 g/L CHN-19 (*Lactococcus lactis subsp. cremoris, L. lactis subsp. lactis, Leuconostoc mesenteroides subsp. cremoris* and *L. lactis subsp. Diacetylactis)* and 0.067 g/L ST-M5 (*Streptococcus thermophiles)* Chymosin (Chymax plus)	120 days at 12 °C	2.5 kg molds	-	Sheehan et al. [12]
Caciocavallo cheeses	-	CM, EM and GM	Pure CM, 4.55CM:1EM and 1.86CM:1GM (v/v) ^1^	-	Innoculum with 1 U/100 L (commercial starter: *Streptococcus thermophilus*, *Lactococcus lactis ssp*. *lactis* and *ssp. cremoris, Lactobacillus helveticus, Lactobacillus casei*, and *Lactobacillus delbrueckii ssp. bulgaricus*), rennet (50 mL/100 L)	60 days at 10 °C with a RH of 80%	2 kg	Pear like shape	Niro et al. [31]
Soft pickled cheese (i.e., Domiati)	Egypt	CaM and BM	Pure CaM, pure BM, 9CaM:1BM, 4CaM:1BM, 2,33CaM:1BM and 1.5CaM:1BM (v/v) ^1^	-	0.04% CaCl_2_ and rennet at a rate of 4 g/100 kg of milk	60 days at 5 °C	-	-	Shahein et al. [32]
Soft cheeses	Sudan	CaM and EM	Pure CaM, pure GM, 3CaM:1GM, 1CM:1EM and 1CM:3EM (v/v) ^1^	-	Camifloc enzyme, calcium chloride, and sodium chloride	21 days in the whey at room temperature (37 °C–40 °C).	-	-	Derar and El Zubeir [33]
White soft cheese (Jibna-beida)	Sudan	CaM and CM	1CaM:1CM (v/v) ^1^	-	Citric acid or LAB starter culture, rennet was added at the rate of 0.15 mL/L of milk	60–120 days at 4 °C	-	Blocks	Siddig et al. [34]
Feta Cheese	Greece	GM and EM	Pure GM, pure EM, 1GM:1EM, 3GM:1EM and 1GM:3EM (v/v) ^1^	-	Yogurt starter culture (*Str. thermophilus* and *L. bulgaricus* 1:1), HALA was added at 0.5% at 37–38 °C and left to ripen for 10 min. CaCl_2_ solution (10%) was added at a rate of 1 mL/kg of milk, followed by the addition of HALA commercial powder rennet.	120 days at 2–3 °C	1.2–1.3 kg	mold (12 × 10 × 10 cm)	Mallatou et al. [35]
Picante cheese	Portugal	GM and EM	Pure GM, pure EM, 1GM:1EM, 3GM:1EM and 1GM:3EM (v/v) ^1^	Goat (Chamequeira breed) ewe (Frfzia breed)	Animal rennet (without addition of a starter culture)	0, 9, 25, 40, 55, 83, 110, 140, and 180 days	-	-	Freitas and Malcata [36]

^1^ v/v: volume/volume; BM: buffalo’s milk; CM: cow’s milk; CaM: camel’s milk; EM: ewe’s milk; GM: goat’s milk; RH: relative humidity.

**Table 2:** Examples of principal conclusions of studies concerning the effect of mixing milk from different species at different proportions on physicochemical and biochemical properties of cheeses ^1^.

Product	Principal Conclusions	Reference
Caciocavallo cheese	Compared to pure CM cheeses, cheeses formulated with 4.55CM:1EM (v/v) presented the highest acidity, while cheeses formulated with 1.86CM:1GM (v/v) presented the highest pH values. Compared to pure CM cheeses, cheeses formulated with 1.86CM:1GM (v/v) had a lower fat content and a higher ash content. The different ratios of CM and EM in mixtures had no significant effect on proteins.	Niro et al. [31], Niro et al. [37]
Crottin de Chavignol cheese	Compared to pure CM, cheeses containing GM (10 to 40%) presented lower moisture, higher fat, and protein contents. Increasing GM ratio in the mixture increased Fat-in-dry matter FDM contents of cheeses.	Chacón-Villalobos and Pineda-Castro [38]
Semi hard cheese	The pH values had no significant differences among the different cheeses milk mixtures (CM and GM). Fat content of 1CM:1GM (v/v) cheese and pure GM cheese were higher than in pure CM. Moisture, protein and salt contents of 1CM:1GM (v/v) cheese and pure GM cheese were lower than in pure CM.	Queiroga et al. [30]
	Increasing CM ratio in cheese made from GM and CM caused an increase in curd pH. Increasing the proportion of CM into GM cheeses increased moisture, fat, and FDM contents. Mixing CM and GM had no significant effect on protein and salt contents. Increasing GM ratio in cheese mixtures tended to increase FFAs contents. The rates of primary proteolysis in terms of nitrogen soluble were not affected by substitution with up to 50% CM. Pure GM and 1CM:1GM (v/v) samples showed higher fatty acids content.	Sheehan et al. [12]
	Increasing GM in cheeses made by mixing GM with CM (1GM:9CM, 1GM:4CM, 1GM:2.33 CM, and 1GM:1.5CM, v/v) increased pH, fat and protein content. Fat-in-dry matter increased according to the amount of GM ratio. No significant differences were observed for lactose and non-fat solids for the different formulations.	Ramirez-Lopez and Vélez-Ruiz [28]
Soft Pickled cheese	The partial substitution of CaM by BM (10 to 40%): ○ increased fat, total solids, protein and lactose contents of cheeses. ○ decreased moisture and ash contents of cheeses.	Shahein et al. [32]
White cheese	Increasing EM ratio into CaM cheeses resulted in increasing acidity values. CaM cheese fortified with EM (25 and 75%) reported an increase in fat and total solids.	Derar and El Zubeir [22]
	Higher moisture content was reported for 1CaM:1CM (v/v) cheeses compared to pure CaM samples.	Siddig et al. [34]
Caciocavallo cheese	Proteolysis was slower in 1.86CM:1GM (v/v) cheeses and faster in pure CM and EM cheeses. The highest TFAA content was found in cheeses made from CM and the lowest in 1.86CM:1GM cheeses. SN/TN value for 1.86CM:1GM (v/v) cheese was higher than pure CM and 4.55CM:1EM (v/v) cheeses. NPN/TN index showed a slower proteolysis in CM:GM cheese and faster proteolysis in CM:EM and CM cheeses.	Niro et al. [37]
Picante cheese	Increasing GM ratio into CM cheese gives rise to increase total concentration of free fatty acid. Increasing CM ratio into GM cheeses increased β-casein and αs-casein degradation and decreased γ-caseins levels.	Freitas and Malcata [36], Freitas and Malcata [39]
Minas fresh cheese	1CM:1GM cheeses displayed higher amounts of cis C18:2n − 6 compared with CM samples. The percentage of C8:0 was higher in 1CM:1GM (v/v) cheeses on d 7 of storage compared with GM cheeses. CM and GM cheeses displayed increased FA content, which was not observed in 1CM:1GM on d 14 of storage. Proteolysis for pure CM cheeses were lower than for 1CM:1GM and GM after 1 day of storage.	Sant’Ana et al. [29]

^1^ v/v: volume/volume; CM: cow’s milk; CaM: camel’s milk; EM: ewe’s milk; GM: goat’s milk.

**Table 3:** Examples of principal conclusions of studies concerning the effect of mixing milk from different species at different proportions on rheological and sensory properties of cheeses ^1^.

Products	Principal Conclusion	Reference
Caciocavallo cheese	L*, a*, b* values monitored on the surface and in the inner part of cheeses, did not show significant changes regarding milk mixtures (CM mixed with EM or GM). Cheeses produced with mixed CM and EM presented higher scores for intensity of flavor, acidic, friability and salty attributes. Cheeses produced with mixed CM and GM displayed higher levels for solubility (meltability of cheese in saliva, intensity of the aroma and bitter attributes. Cheeses produced with mixed CM to GM tended to improve sensory properties of cheeses. Adding GM or EM into CM improved sensory characteristics of Caciocavallo cheese (texture, flavor and aroma).	Niro et al. [31]
Feta cheese	Increasing GM ratio in cheeses tended to weaken them and increased the force needed to fracture the sample. Cheeses with higher proportion of GM exhibited high level of hardness. Pure EM cheese received higher body-texture scores than the other cheeses (1GM:1EM (v/v), 1GM:3EM (v/v), 3GM:1EM (v/v), and pure GM). No significant differences were observed in terms of flavor among cheeses made from mixed GM and EM.	Mallatou et al. [35]
Minas fresh cheese	1CM:1GM (v/v) cheeses displayed higher hardness (*p* < 0.05) than CM and GM samples. 1CM:1GM (v/v) and CM cheeses displayed decreased (*p* < 0.05) gumminess and chewiness values compared to GM. 1CM:1GM (v/v) cheeses promoted a decrease (*p* < 0.05) in creamy color and an increase in the white color compared with other samples. The cheeses evaluated did not differ (*p* > 0.05) in their smooth appearance, soft and homogeneous texture, or salty and acidic flavor.	Sant’Ana et al. [29]
Caolho cheese	Mixing CM and GM had no effect on cheeses’ chewiness and cohesiveness. 1CM:1GM (v/v) and GM cheeses presented higher whiteness values (L*) from 7 days of storage.	Queiroga et al. [30]
Semi hard cheese	Mixing CM with GM did not affect whiteness of cheeses. Cheese yellowness was dependent on the ratio of milk type (GM/CM, v/v). Partial substitution of GM with CM in cheeses with 25% or greater levels increased cheese greenness.	Ramirez-Lopez and Vélez-Ruiz [28], Sheehan et al. [12]
Picante cheese	Mixing EM and GM did not affect cheese sensory properties (surface, form, texture, and flavor).	Freitas et al. [40]

^1^ v/v: volume/volume; CM: cow’s milk; CaM: camel’s milk; EM: ewe’s milk; GM: goat’s milk.
